# Microbial sulfur transformations in sediments from Subglacial Lake Whillans

**DOI:** 10.3389/fmicb.2014.00594

**Published:** 2014-11-19

**Authors:** Alicia M. Purcell, Jill A. Mikucki, Amanda M. Achberger, Irina A. Alekhina, Carlo Barbante, Brent C. Christner, Dhritiman Ghosh, Alexander B. Michaud, Andrew C. Mitchell, John C. Priscu, Reed Scherer, Mark L. Skidmore, Trista J. Vick-Majors, the WISSARD Science Team

**Affiliations:** ^1^Department of Microbiology, University of TennesseeKnoxville, TN, USA; ^2^Department of Biological Sciences, Louisiana State UniversityBaton Rouge, LA, USA; ^3^Climate and Environmental Research Laboratory, Arctic and Antarctic Research Institute, St.Petersburg, Russia; ^4^Institute for the Dynamics of Environmental Processes – Consiglio Nazionale delle Ricerche and Department of Environmental Sciences, Informatics and Statistics, Ca’ Foscari University of VeniceVenice, Italy; ^5^Department of Land Resources and Environmental Sciences, Montana State UniversityBozeman, MT, USA; ^6^Geography and Earth Sciences, Aberystwyth UniversityCeredigion, UK; ^7^Department of Geological and Environmental Sciences, Northern Illinois UniversityDeKalb, IL, USA; ^8^Department of Earth Sciences, Montana State UniversityBozeman, MT, USA

**Keywords:** Antarctic subglacial aquatic environments, geomicrobiology, chemosynthesis, sulfur oxidation, sulfate reduction

## Abstract

Diverse microbial assemblages inhabit subglacial aquatic environments. While few of these environments have been sampled, data reveal that subglacial organisms gain energy for growth from reduced minerals containing nitrogen, iron, and sulfur. Here we investigate the role of microbially mediated sulfur transformations in sediments from Subglacial Lake Whillans (SLW), Antarctica, by examining key genes involved in dissimilatory sulfur oxidation and reduction. The presence of sulfur transformation genes throughout the top 34 cm of SLW sediments changes with depth. SLW surficial sediments were dominated by genes related to known sulfur-oxidizing chemoautotrophs. Sequences encoding the adenosine-5′-phosphosulfate (APS) reductase gene, involved in both dissimilatory sulfate reduction and sulfur oxidation, were present in all samples and clustered into 16 distinct operational taxonomic units. The majority of APS reductase sequences (74%) clustered with known sulfur oxidizers including those within the “Sideroxydans” and* Thiobacillus* genera. Reverse-acting dissimilatory sulfite reductase (rDSR) and 16S rRNA gene sequences further support dominance of “Sideroxydans” and *Thiobacillus* phylotypes in the top 2 cm of SLW sediments. The SLW microbial community has the genetic potential for sulfate reduction which is supported by experimentally measured low rates (1.4 pmol cm^-3^d^-1^) of biologically mediated sulfate reduction and the presence of APS reductase and DSR gene sequences related to *Desulfobacteraceae* and *Desulfotomaculum*. Our results also infer the presence of sulfur oxidation, which can be a significant energetic pathway for chemosynthetic biosynthesis in SLW sediments. The water in SLW ultimately flows into the Ross Sea where intermediates from subglacial sulfur transformations can influence the flux of solutes to the Southern Ocean.

## INTRODUCTION

Subglacial aquatic environments exist beneath the Antarctic Ice Sheet as lakes, streams, marine brines, and water-saturated sediments (Priscu et al., 2008; Fricker and Scambos, 2009; Mikucki et al., 2009; Skidmore, 2011; Wright and Siegert, 2012). Recently, the Whillans Ice Stream Subglacial Access Research Drilling (WISSARD) project directly sampled water and sediment from Subglacial Lake Whillans (SLW), one of the 379 identified Antarctic subglacial lakes (Wright and Siegert, 2012). Initial analyses of samples collected from SLW show the presence of an active community of diverse heterotrophic and autotrophic microorganisms in the water column and surficial sediments (Christner et al., 2014). Substrates for subglacial growth are primarily derived from minerals and organic matter in the underlying sediments (Tranter et al., 2005). Recent evidence for the presence of sulfur cycling microorganisms in Antarctic subglacial environments (Christner et al., 2006, 2014; Lanoil et al., 2009; Mikucki et al., 2009), implies that sulfur transformations may provide chemical energy for growth in these cold, dark ecosystems. Measurements of metabolic substrate concentrations, enrichment cultures, molecular surveys, sulfur and oxygen isotopic composition, and microcosm experiments have been used to infer the presence of sulfate reduction and sulfide oxidation beneath Arctic and Alpine glaciers (Skidmore et al., 2000, 2005; Bottrell and Tranter, 2002; Wadham et al., 2004; Montross et al., 2013). Similarly in Antarctic subglacial systems, sulfide oxidation has been inferred as an important microbial process from 16S rRNA gene sequence data (Mikucki and Priscu, 2007; Lanoil et al., 2009) and geochemical measurements (Skidmore et al., 2010; Wadham et al., 2010). However, there have been few studies on functional gene diversity in Antarctic subglacial systems.

Sulfur is required for cellular components, such as the amino acids cysteine and methionine; however, some microorganisms utilize sulfur compounds in dissimilatory, energy-yielding metabolic processes. The sulfur-oxidizing prokaryotes (SOP) are metabolically and phylogenetically diverse (Friedrich et al., 2001, 2005), and can fix CO_2_ utilizing a variety of electron acceptors including O_2_, NO_3_^-^, Mn^3+/4+^, and Fe^3+^ (Mattes et al., 2013). Sulfate-reducing prokaryotes (SRP) respire organic material for energy and use sulfate as an electron acceptor when oxygen is absent (Jørgensen, 1982; Jørgensen and Postgate, 1982). Reduced sulfur compounds generated by sulfate reduction can provide energy for the SOP component of the community, although a larger fraction of reduced sulfur for microbial oxidation may come from mineral sources. This may be important subglacially, where the grinding of glacial ice over bedrock would expose reactive mineral surfaces (Anderson, 2007). Sulfate reduction is widely recognized as an important process in other dark and cold environments including anaerobic marine sediments, where it contributes to greater than 50% of total organic carbon oxidation globally (Canfield et al., 1993; Thullner et al., 2009; Bowles et al., 2014). The linkage of the sulfur and carbon cycles in Antarctic subglacial environments has not been well characterized. Here we aimed to survey the potential role of sulfur transforming communities in SLW sediments.

We measured rates of sulfate reduction and analyzed the presence and diversity of three dissimilatory sulfur cycling genes (APS, DSR, and rDSR) in sediments collected from SLW. APS reductase is a conserved enzyme among both SRP and SOP (Meyer and Kuever, 2007c) and the alpha subunit of APS reductase, *aprA*, is a common marker for both metabolic groups (Meyer and Kuever, 2007c). DSR is found in all SRP and catalyzes the final energy-yielding step of sulfite reduction to hydrogen sulfide (Wagner et al., 1998; Zverlov et al., 2005; Rabus et al., 2006). A homolog of DSR, reverse-acting DSR (rDSR), is a marker for some sulfur-storing and oxidizing members of the phyla Chlorobi and Proteobacteria and is thought to be involved in the oxidation of intracellular stored elemental sulfur compounds (Pott and Dahl, 1998; Loy et al., 2008, 2009).

Our results show that prokaryotes in SLW sediments mediate sulfur transformations and that the potential for sulfur oxidation by chemosynthetic bacteria is present in SLW sediments. Microbial sulfur metabolism can influence mineral dissolution and precipitation indirectly via production of acidic metabolic byproducts, or directly via electron transfer (Ehrlich, 1996; Banfield et al., 1999). Because water beneath the WIS drains into the surrounding ocean (Carter and Fricker, 2012), microbial transformations of sulfur beneath the WIS could influence biogeochemical cycling upon release of metabolic byproducts into the Ross Sea and possibly the Southern Ocean.

## MATERIALS AND METHODS

### SITE DESCRIPTION AND SAMPLE COLLECTION

**FIGURE 1:**
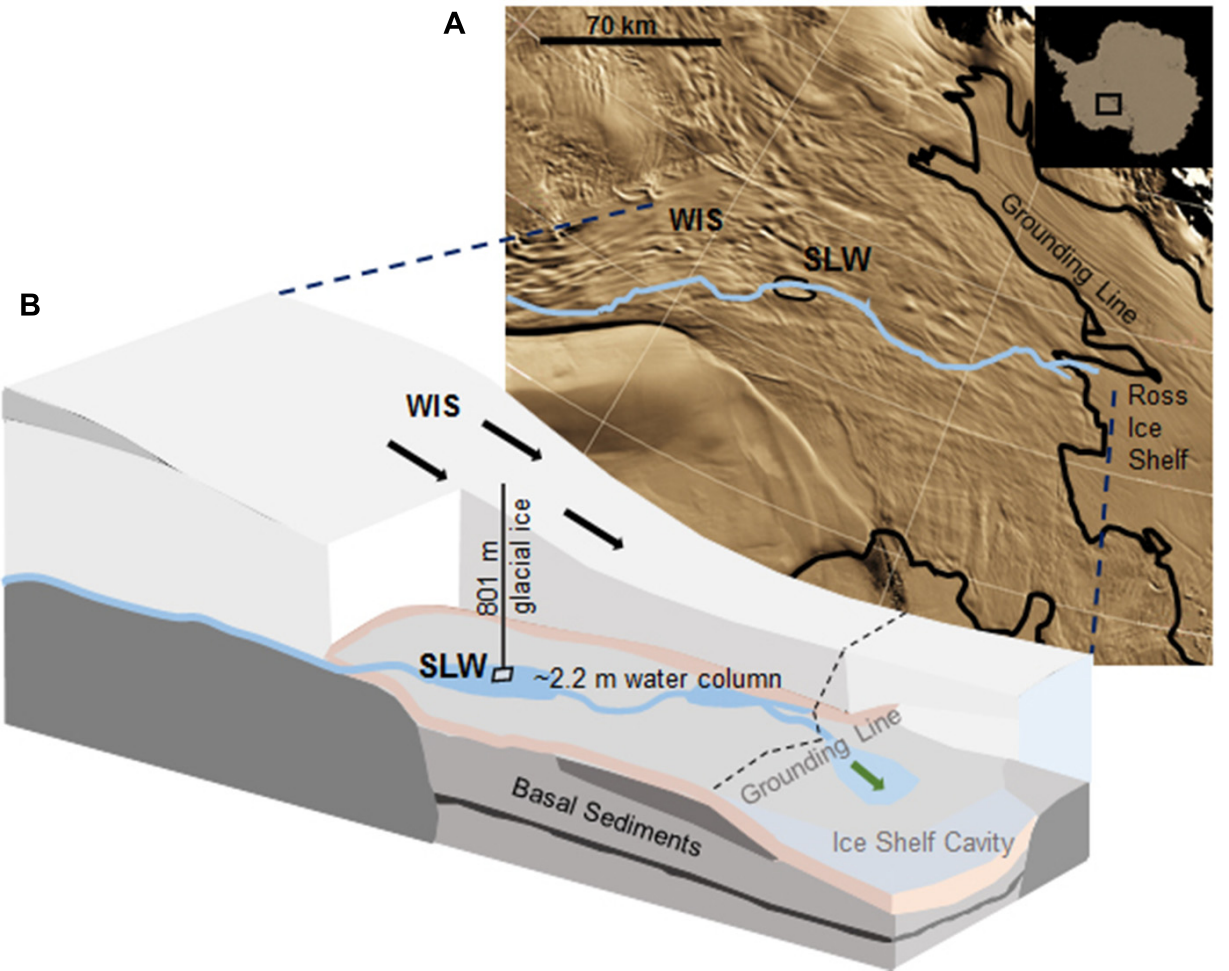
**Location of Subglacial Lake Whillans (SLW) and schematic of the Whillans Ice Stream (WIS). (A)** Satellite image of the Siple Coast with SLW labeled (after Fricker and Scambos, 2009); the blue line indicates the proposed subglacial water flow path toward the grounding line (Carter and Fricker, 2012). Background satellite image from MODIS Mosaic of Antarctica (Haran et al., 2005). **(B)** Cross-sectional cartoon of the WIS indicating the borehole created through 801 m of ice. Sediment cores were collected through ∼2.2 m of water. Black arrows indicate the direction of ice movement; green arrow indicates predicted dispersal of subglacial water into the marine cavity beneath the Ross Ice Shelf. Cross-sectional cartoon of the WIS adapted from Fricker et al. (2011).

Subglacial Lake Whillans is located beneath the downstream portion of the WIS (S 84.237°, W 153.614°; Christianson et al., 2012), ca. 100 km from the grounding zone, where the ice sheet transitions into the Ross Ice Shelf (**Figure 1**). SLW is a shallow (∼2.2 m deep; Tulaczyk et al., 2014) lake located in what appears to be a large wetland along the Siple Coast of West Antarctica (Priscu et al., 2010; Fricker et al., 2011). SLW is considered an ‘active’ lake as it drains and refills on a sub-decadal time scale discharging water towards the Ross Sea (Fricker et al., 2007; Carter and Fricker, 2012; Siegfried et al., 2014). In January 2013, the WISSARD Project^1^ used hot water drilling to penetrate 801 ± 1 m of glacial ice to access SLW. Details of drilling operations are described elsewhere (Tulaczyk et al., 2014). A clean access protocol (Priscu et al., 2013) was followed to maintain both sample integrity and environmental stewardship. Briefly, drilling water was passed through two filtration units (2.0 and 0.2 μm) to remove large particulates and microbial cells. Water was then subjected to ultraviolet irradiation of 185 nm for organic matter destruction and germicidal 254 nm. Finally, drilling water was pressurized and heated to 90°C and used to melt an access borehole. Instruments were cleaned with 3% hydrogen peroxide and cables and hoses were deployed through a UV collar during deployment down the borehole (Priscu et al., 2013; Christner et al., 2014).

Sediments from SLW were collected using a gravity driven multi-corer (Uwitec) built for recovery of sediment cores and water from the sediment-water interface. The multi-corer successfully recovered ∼40 cm of sediment and 20 cm of basal water in most deployments (Tulaczyk et al., 2014). Sediment cores analyzed in this study were collected from the second (identified as core ‘MC-2B’) and third multicore deployment (core ‘MC-3C’; Tulaczyk et al., 2014). Cores contained conspicuous bubbles when brought to the surface, suggesting possible degassing during core retrieval. Ice above the lake moved ∼5 cm during the second and third multi-core casts, thus samples may represent overlapping locations (Tulaczyk et al., 2014). Approximately two thirds of the sediments from MC-3C slipped out of the core tube, leaving the top ∼16 cm of sediment, which was structurally undisturbed. Cores were vertically extruded and serially sectioned using a core stand and cutter (Uwitec) in a Class 100 laminar flow hood. Sediments were sampled from three depth intervals in each core. MC-2B was sampled at depths of 0–4, 4–8, and 28–34 cm and MC-3C was sampled at depths of 2.0–3.5, 3.5–8.0, and 8–16 cm (**Table 1**). Samples are referred to by core name with the depth in subscript throughout this manuscript, for example MC-2B_(0-4__cm)_. Sediments for metabolic activity experiments were processed on site. Samples for nucleic acid extraction were stored in sterile whirl-pak (Nasco) bags at -10°C at the field site and then shipped to the University of Tennessee in the dark at -20°C.

**Table 1:** Subglacial Lake Whillans sediment samples used in this study, gene amplifications, sulfate reduction rates (SRR), and quantitative-PCR gene quantification.

Sample		Gene				Sulfate Reduction Rates (SRR; pmol cm^-3^ d^-1^)^b^		Gene quantification (copies g^-1^ wet sediment)			
Core	Depth (cm)	*aprA* (384 bp)	*dsrAB* (1.9 kb)	*dsrA* (221 bp)	*rdsrAB*^a^ (1.9 kb)	Without formate	With formate	Bacterial 16S rRNA	Archaeal 16S rRNA	*aprA*	% *aprA* of 16S rRNA^c^
MC-2B	0–4	+	-	+	+	0.42**	0.41**	3.9 × 10^6^	2.4 × 10^6^	9.12 × 10^5^	14.5/23.6
MC-2B	4–8	+	-	+	-	ND	ND	2.6 × 10^4^	5.8 × 10^5^	9.60 × 10^3^	1.6/36.6
MC-2B	28–34	+	+	+	-	ND	ND	1.2 × 10^5^	1.4 × 10^6^	8.42 × 10^3^	0.6/7.3
											
MC-3C	2–3.5	+	-	+	+	1.67*	1.29*	8.5 × 10^6^	4.4 × 10^6^	9.58 × 10^5^	7.4/11.3
MC-3C	3.5–8	+	+	+	+	1.20*	1.84**	2.2 × 10^5^	9.0 × 10^5^	2.47 × 10^4^	2.2/11.1
MC-3C	8–16	+	+	+	-	ND	ND	2.0 × 10^4^	4.0 × 10^5^	3.67 × 10^3^	0.9/18

*Gene amplifications from SLW sediments: “+” indicates positive amplification. “-” indicates no amplification.*

*^a^At least one of the primer combinations amplified (**Table 2**).*

^b^*p* value calculated by one tailed unpaired t-test, NS = not significant when compared with kills; * = significant (p < 0.05); ** = highly significant (*p* < 0.01); The water column was NS. ND = Not Determined.

*^c^First number is % aprA with respect to total prokaryote 16S rRNA gene copies and second is with respect to total bacterial 16S rRNA from Q-PCR quantification.*

### ^35^S-SULFATE INCUBATION EXPERIMENTS

Biologically mediated sulfate reduction was measured using the passive extraction method (Ulrich et al., 1997) following incubation with ^35^SO_4_^2-^ tracer. Sediment (∼5 g) from selected depths (**Table 1**) was aseptically transferred using a sterile spatula into pre-weighed, pre-combusted, N_2_-gassed serum vials. These depths were selected because they corresponded to the lowest reduction potential in both cores (at ∼3.5 cm; Mitchell and Mikucki, unpublished data). MC-2B_(0-4__cm)_ corresponded to the surficial sediments selected for extensive biogeochemical characterization (i.e., Christner et al., 2014). Sulfate reduction experiments were also conducted on SLW lake water (5 ml). All solutions used in this experiment were N_2_-flushed. Sediment slurries were made with the addition of one ml sterile DNA-free water (Fisher) to minimize issues caused by potential isotope diffusion within the sediments. Small test tubes containing 2.5 ml of 10% zinc acetate (sulfide traps) flushed with N_2_ gas were added to each serum vial. Blank serum vials containing sterile water were incubated along with all samples to correct for possible background transfer of the radiolabel to the traps. 2.75 μCi ^35^S-SO_4_^2-^ (specific activity ∼1490 Ci/mmol) was added with a sterile syringe. This injection added 25 nM of sulfate to the porewater. Each sediment sample depth included three live and three killed controls whereas bulk SLW water included four live and four killed samples (kills = 2% paraformaldehyde, final concentration). Because two organic carbon atoms are oxidized for every sulfate ion reduced, formate (50 mM, final concentration) was added to a parallel set of sediment samples to ensure that that organic carbon was present at saturating levels during the incubation period. Samples were incubated at 1–2°C for 9 days. Experiments were terminated with the addition of 6 M HCl (8 ml) and 1M CrCl_2_ in 0.5 M HCl (8 ml). Vials were mixed at 125 RPM for 48 h to ensure all total reactive inorganic S (TRIS) was liberated as H_2_S and precipitated in the zinc traps. This passive extraction method has been shown to efficiently extract TRIS as FeS, FeS_2_, and S^2-^ but has low efficiency for the extraction of S° thus it may underestimate total sulfate reduction (Ulrich et al., 1997). Zinc traps were then removed and the contents added to scintillation cocktail (Cytoscint ES). Activity was measured using standard liquid scintillation spectrometry in the Crary Lab at McMurdo Station. Sulfate reduction rates (SRR; pmol SO_4_^-2^ cm^-3^ d^-1^) were computed according to the equation (Fossing and Jørgensen, 1989)

$$SRR=\frac{a}{A+a}*\frac{\left[SO_{4}^{2-}\right]}{t}*1.06$$

Where *a* is the radioactivity (dpm_live_–dpm_kills_) in the TRIS fraction, *A* is the radioactivity (dpm) added to the sample as ^35^S-SO_4_^2-^. [SO_4_^2-^] is the concentration of sulfate (pmol cm^-3^) in the sample at time zero, *t* is the incubation time (days), and 1.06 is a correction factor for enzymatic isotope discrimination. Sulfate reduction rates presented represent the mean (± SD) of three replicates. Sulfate was present in the water column (0.56 mM) and surficial sediments (0.62 mM; Christner et al., 2014). Density, porosity, and sulfate concentrations in sediment porewater for the experimental depths were based on values collected from the replicate cores, MC-2A (density and porosity) and MC-3B (sulfate concentrations) obtained during the second and third multi-corer casts, respectively (Michaud and Priscu, in preparation). Porosity and density in core MC-2A were measured as described by Riedinger et al. (2010). Given that we imposed anaerobic conditions during these experiments and the exact oxygen levels *in situ* remain unknown, all rates should be considered as potential.

### MICROBIAL CELL ENUMERATION

Cells were enumerated from one sediment sample collected from the exterior of an instrument that penetrated into the sediments to a depth no greater than 20 cm; therefore, this sample was not obtained from a discrete depth. Cells were extracted from bulk sediments as follows. Slurries were prepared in triplicate by homogenizing sediments (2–4 g) with 1X PBS buffer (final ratio 1:2). Slurries were fixed with paraformaldehyde (2% final concentration) for ∼16 h, then methanol and a 1% Tween80 solution were added (10% final concentration) to detach cells from sediments; this chemical extraction step was modified from Kallmeyer et al. (2008). Slurries were vortexed at medium–high speed at 4°C for 30 min. then centrifuged at a low speed, 50 × *g*, to settle out the coarser sediment particles (at least 1 h at 4°C). The supernatant (2–3 mls), including the cells and the finest sediment fraction was then filtered onto a 0.2 μm polycarbonate filter and stained with 25X SYBR Gold nucleic acid stain (Invitrogen^TM^) for 15 min (Ball and Virginia, 2014). Filters were rinsed with 1 ml of 0.2 μm filtered nanopure water and enumerated using epi-fluorescence microscopy (Leica DM5500B with an excitation filter set BP 480/40). A procedural blank using the solutions was processed alongside the sample replicates and quantified to assess any potential contamination. Three ml of autoclaved 0.2 μm-filtered nanopure water was passed through the filtration towers and filtered before each sample replicate and quantified and subtracted from total sample counts. Our extraction method required ∼6 g of sediment, which limited our ability to analyze each depth in our study. A quantitative-PCR (Q-PCR) approach was used instead to estimate abundance despite known caveats, including PCR inhibitors in environmental samples, and DNA extraction and primer biases (Smith and Osborn, 2009).

### DNA EXTRACTION AND PCR AMPLIFICATION

DNA was extracted in triplicate from 0.3–0.4 g of sediment in a class II type A2 clean hood (LabConco model #3460001) using the FastDNA^TM^ SPIN Kit (MP Biomedicals) according to the manufacturer’s protocol. Eluent containing DNA from each replicate was pooled. Kit solutions were extracted simultaneously as a control for methodological contamination. A fragment (384–396 bp) of the alpha subunit of *aprA* was amplified using the forward primer AprA-1-FW and reverse primer AprA-5-RV (Meyer and Kuever, 2007c). A short fragment (221 bp) of the alpha subunit of *dsrA* was amplified using forward primer DSR1F+ and reverse primer DSR-R (Kondo et al., 2004). DSR alpha and beta subunits (*dsrAB*) were amplified (1.9 kb) using forward primer DSR1F and reverse primer DSR4R (Wagner et al., 1998). Reverse dissimilatory reductase alpha and beta subunits (*rdsrAB*) were amplified with all published forward and reverse *rdsrAB* primer combinations (Loy et al., 2009; Lenk et al., 2011). All primer sequences are listed in **Table 2**. REDTaq^®^ ReadyMix^TM^ PCR Reaction Mix (Sigma Aldrich) was used with each primer combinations according to the manufacturer’s protocol. PCR reactions contained 25 μl RedTaq, 4 μl of template (< 15 ng DNA ul^-1^), 1 μl of each forward and reverse primer (final primer concentration 200 nM), and 19 μl of nuclease-free water for a final volume of 50 μl. Amplification of *aprA* was initiated at 94°C for 2 min, followed by 40 cycles (Green-Saxena et al., 2012) of 1 min 94°C denaturation; 1 min 48°C annealing; and 1 min 72°C extension, then a final elongation of 7 min at 72°C. Amplification of *aprA* was increased to 43 cycles for core MC-2B_(4-8__cm)_ because no amplification was observed after 40 cycles. Annealing temperature was increased to 57°C and repeated for 41 cycles for *dsrA* amplification. *dsrAB* and *rdsrAB* amplification was performed as described for *aprA*, but included 42 cycles, 2 min extension, with a final elongation of 7 min Extracts (4 μl) from kit blanks and sterile water reagent blanks were processed for PCR controls for all amplifications. No amplification products were detected in the controls without template or in extraction kit blanks.

**Table 2:** DNA oligonucleotide primers used in this study.

Primer and use	Sequence (5’–3’)	Reference
**PCR Amplification and cloning**		
AprA-1-FW Forward	TGGCAGATCATGATYMAYGG	Meyer and Kuever (2007c)
AprA-5-RV Reverse	GCGCCAACYGGRCCRTA	Meyer and Kuever (2007c)
DSR1F+ Forward	ACSCACTGGAAGCACGGCGG	Kondo et al. (2004)
DSR-R Reverse	GTGGMRCCGTGCAKRTTGG	Kondo et al. (2004)
DSR1 Forward	ACSCACTGGAAGCACG	Wagner et al. (1998)
DSR4 Reverse	GTGTAGCAGTTACCGCA	Wagner et al. (1998)
rDSR1Fa	AARGGNTAYTGGAARG	Loy et al. (2009)
rDSR1Fb	TTYGGNTAYTGGAARG	Loy et al. (2009)
rDSR1Fc	ATGGGNTAYTGGAARG	Loy et al. (2009)
rDSR4Ra	CCRAARCAIGCNCCRCA	Loy et al. (2009)
rDSR4Rb	GGRWARCAIGCNCCRCA	Loy et al. (2009)
rDSRA240F	GGNTAYTGGAARGGNGG	Lenk et al. (2011)
rDSR808R	CCDCCNACCCADATNGC	Lenk et al. (2011)
**Sequencing**		
T3	ATTAACCCTCACTAAAGGGA	
T7	TAATACGACTCACTATAGGG	
**Q-PCR**		
Bac340 Forward	TCCTACGGGAGGCAGCAGT	Nadkarni et al. (2002)
Bac515 Reverse	CGTATTACCGCGGCTGCTGGCAC	Nadkarni et al. (2002)
Arc915 Forward	AGGAATTGGCGGGGGAGCAC	Takai and Horikoshi (2000)
Arc1059 Reverse	GCCATGCACCWCCTCT	Yu et al. (2005)

### QUANTITATIVE PCR

Gene copy abundances of bacterial and archaeal 16S rRNA genes and *aprA* were measured in triplicate (technical replicates) using an iQ^TM^5 Multicolor Real-Time PCR Detection System (Bio Rad). Standards for bacterial and archaeal 16S rRNA genes were constructed using DNA extracted from pure cultures of *Escherichia coli* and *Methanocaldococcus jannaschii*, respectively. 16S rRNA genes were amplified then gel purified using Wizard PCR clean up (Promega), and cloned using the TOPO^®^ TA cloning kit (Life Technologies) with One Shot^®^ TOP10 Chemically Competent *E. coli* and the pCR^TM^4 cloning vector. Plasmids were purified using the PureYield^TM^ Plasmid Miniprep System (Promega). Starting gene copy abundances in extracted plasmid were calculated according to Ritalahti et al. (2006). Plasmids were serially diluted to concentrations of 1 × 10^1^ to 1 × 10^9^ copies μl^-1^. *aprA* gene standards were made using plasmids extracted from an *aprA* clone from this study and were diluted to concentrations of 1 × 10^1^ to 1 × 10^7^ copies μl^-1^. A two-step protocol described by Lloyd et al. (2011) was used to quantify amplification under the following conditions: 95°C for 5 min and 40 cycles of 95°C for 1 min and 60°C for 30 sec Melting curves were performed at 0.5°C steps from 55°C to 95°C and analyzed after each quantification to check for primer dimer formation and amplification specificity. All samples were quantified on the same Q-PCR run and samples from the same DNA extraction were used to quantify all genes. 16S rRNA primer targets and coverage were checked *in silico* using TestPrime 1.0 application (Klindworth et al., 2012) using the SILVA SSU r119 RefNR database (Quast et al., 2013^2^). 16S rRNA gene primer sets in this study (**Table 2**) cover 73% of the domain Bacteria (0% Archaea) and 73% of the domain Archaea (0% Bacteria). Primers from this study (**Table 2**), have successfully been used for *aprA* quantification in Peru margin sediment samples (Blazejak and Schippers, 2011). All reactions had a final volume of 25 and 12.5 μl of QuantiFast SYBR Green PCR mastermix (Qiagen, Valencia, CA), and 2 μl of template. Primer concentrations were 80 nM for bacterial and archaeal 16S rRNA genes (Lloyd et al., 2011) and 200 nM for *aprA*. All standards were only thawed once and run in triplicate. Threshold cycles were averaged to make a standard curve. Starting quantities were calculated from a log-linear standard curve (R^2^ value ≥ 0.98). The instrument detection limit for archaeal 16S rRNA genes were 1 × 10^3^ copies μl^-1^, bacterial 16S rRNA genes were 1 × 10^2^ copies μl^-1^, and *aprA* was 1 × 10^1^ copies μl^-1^. Controls included DNA extraction blanks and Q-PCR reagents without template. Controls amplified more than 2 threshold cycles later than samples or fell below the quantification limit for each gene.

### CLONE LIBRARY CONSTRUCTION

Amplicons of *aprA*, *dsrA*, *dsrAB*, and *rdsrAB* genes were purified by gel extraction using the Wizard^®^ SV Gel and PCR Clean-Up System (Promega) following the manufacturer’s protocol. Products from 7 primer sets for *rdsrAB* were pooled; for all other genes, only one primer set was used (**Table 2**). Clone libraries were constructed using TOPO^®^ TA cloning kit (Invitrogen^TM^) with One Shot^®^ TOP10 Chemically Competent *E. coli* and the pCR^TM^4 cloning vector. Approximately 50 colonies were randomly picked after growth on LB agar plates with kanamycin and cultured in LB. Plasmids were extracted using the PureYield^TM^ Plasmid Miniprep System (Promega) following the manufacturer’s protocol. Sanger sequencing was performed on the extracted plasmids using the T3 and T7 primers (**Table 2**). ABI Big-Dye v3.1 cycle sequencing mix was used for reactions run on an ABI 3130 analyzer (Applied Biosystems) at the University of Tennessee, Knoxville Molecular Biology Resource Facility and the Clemson University Genomics Institute.

### PHYLOGENETIC AND DIVERSITY ANALYSES

Nucleotide sequences were imported into BioEdit version 7.2.3 (Hall, 1999^3^). Vector sequence was removed, and nucleotide sequences were checked for possible chimeric artifacts using the Bellerophon program (Huber et al., 2004) or manually by BLASTn alignment analysis (Antony et al., 2010; Nilsson et al., 2010). Nucleotide sequences were translated into amino acid sequences and aligned using ClustalW (Larkin et al., 2007) in BioEdit (Hall, 1999). Numerous functional gene diversity studies of environmental samples studies use an amino acid sequence identity cut off 90–97% to describe distinct operational taxonomic units (OTUs; Loy et al., 2009; Lenk et al., 2011; Leloup et al., 2009). Here we define OTUs for *aprA*, *dsrA*, and *rdsrA* sequences as clusters of sequences having an amino acid sequence identity of 90% or greater (Loy et al., 2009; Lenk et al., 2011). All functional gene sequences in this study were grouped into OTUs using the BlastClust tool^4^. Only the alpha subunit portion of the 1.9 kb *dsrAB* and *rdsrAB* (255 amino acids) was used for cluster analyses. Amino acid sequences were searched against the NR database in NCBI using BLASTp^5^.

Phylogenetic analysis included aligning *aprA* sequences from reference strains and sequences of highest amino acid identity to cultured and uncultured *aprA* sequences in the NR database. *aprA* OTUs were then functionally classified as SRP or SOP based on lineages defined by Meyer and Kuever (2007a). These lineage designations are supported by amino acid indels that are unique to the lineages (Meyer and Kuever, 2007a,b). Sequences that did not fall within either the SRP or SOP *aprA* lineages were referred to as sequences of uncertain function. After designating *aprA* sequences into OTUs, one representative sequence from each OTU was selected for tree construction. A neighbor-joining tree was constructed using MEGA version 6 (Tamura et al., 2013) and Jones-Thornton-Taylor (JTT) substitution model and a bootstrap analysis of 1000 replicates.

Statistical tests (**Table 3**) were calculated to evaluate *aprA* diversity including, Shannon-Weaver diversity index (*H′)* and Simpson’s index (*D*). Sampling coverage was evaluated using the Chao1 richness estimator, Good’s coverage, and rarefaction. Good’s coverage (*C*) was calculated using the equation *C = 1–(n_i_/N)*, where *n_i_* is the number of single unique clones, and *N* is the total number of clones in the library (Good, 1953; Singleton et al., 2001). Simpson’s and Shannon-Weaver diversity indices, and Chao1 richness estimator were calculated using standard equations in Hill et al. (2003). Chao1 richness estimator was calculated to estimate the OTU abundance expected in each clone library using standard equations in Hill et al. (2003). Rarefaction curves were generated to estimate the thoroughness of sequencing. Curves were generated using R version 2.15.0 (R Development Core Team, 2008) and a modified script^6^ to evaluate our clone library size.

**Table 3:** Estimates of *aprA* diversity, richness, and clone library coverage in SLW sediments.

Sediment sample	All	MC-2B_(0-4 cm)_	MC-2B_(4-8 cm)_	MC-2B_(28 34cm)_	MC-3C_(2-3.5 cm)_	MC-3C_(3.5-8 cm)_	MC-3C_(8-16 cm)_
Total # clones	275	45	28	39	45	40	39
Total # operational taxonomic unit (OTUs)	16	6	3	8	4	6	8
Good’s coverage	0.98	0.93	0.96	0.92	0.98	0.93	0.92
Simpson’s index (D)	0.40	0.61	0.5	0.24	0.68	0.45	0.16
Shannon–Weaver index (H′)	1.50	0.83	0.77	1.62	0.63	1.09	1.82
Chao1 richness estimator	34	11	3	13	5	6	8

### NUCLEOTIDE SEQUENCE ACCESSION NUMBERS

Nucleotide sequences of the *aprA, dsrA, and rdsrA* genes from this study were deposited in GenBank under the accession numbers KM589857–KM590347.

## RESULTS

### ACTIVITY OF SULFATE-REDUCING PROKARYOTES

**FIGURE 2:**
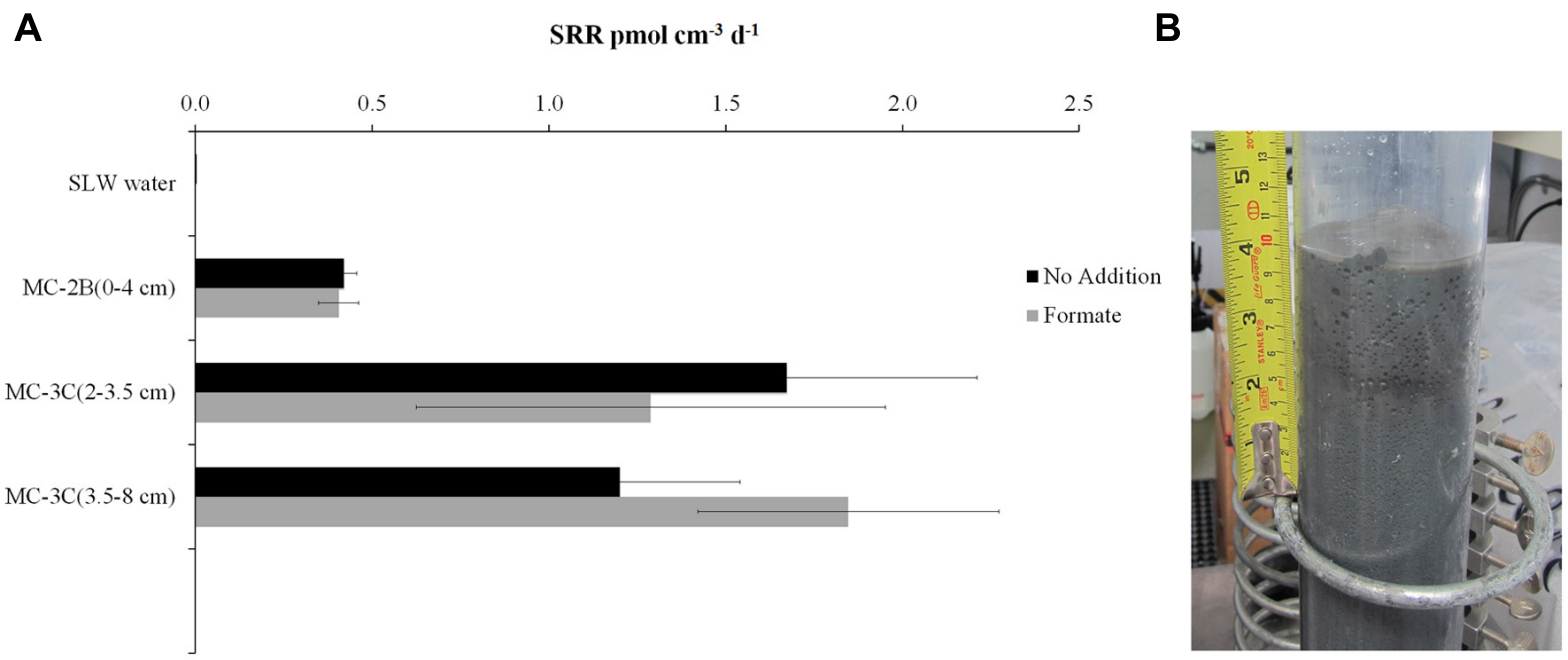
**Sulfate reduction rates (SRR) in SLW sediment samples. (A)** SRR for killed controls was subtracted from each sample replicate. Black bars represent sediment incubations with no carbon addition; Gray bars represent sediment incubations with 50 mM formate addition (± SD of triplicates). **(B)** Image of SLW sediment core MC-2B.

^35^SO_4_^2-^ amended incubation experiments of sediment slurries indicated that SRR were statistically significant in all three of the SLW sediments samples tested, albeit at low rates (average = 1.4 pmol cm^-3^d^-1^ ± 0.60; **Table 1**; **Figure 2**). There was no significant stimulation in sulfide production with the addition of formate (**Table 1**; **Figure 2**). Activity in SLW water column samples was not detected (i.e., live samples were not statistically greater than kills). The highest rates of sulfate reduction were measured in unamended samples from MC-3C_(2-3.5__cm)_ (avg = 1.7 pmol cm^-3^ d^-1^ ± 0.54) and formate amended MC-3C_(3.5-8__cm)_ (avg = 1.8 pmol cm^-3^d^-1^ ± 0.43; **Table 1**; **Figure 2**). Lower rates were measured in MC-2B_(0-4__cm)_ (avg = 0.4 pmol cm^-3^ d^-1^ ± 0.04; **Table 1**; **Figure 2**). MC-3C was stored at 4°C in its core tube for ∼24 h while MC-2B was processed; however, the sediments collected from MC-2B were likely exposed to oxygen during processing. Unlike the MC-3C samples for SRR, MC-2B sediments were not immediately transferred to N_2_-gassed serum vials due to logistical constraints. Thus, variations in rates between depths could have been affected by limitations of our field laboratory.

### QUANTIFICATION OF BIOMASS AND TOTAL 16S rRNA AND *aprA* GENES

**FIGURE 3:**
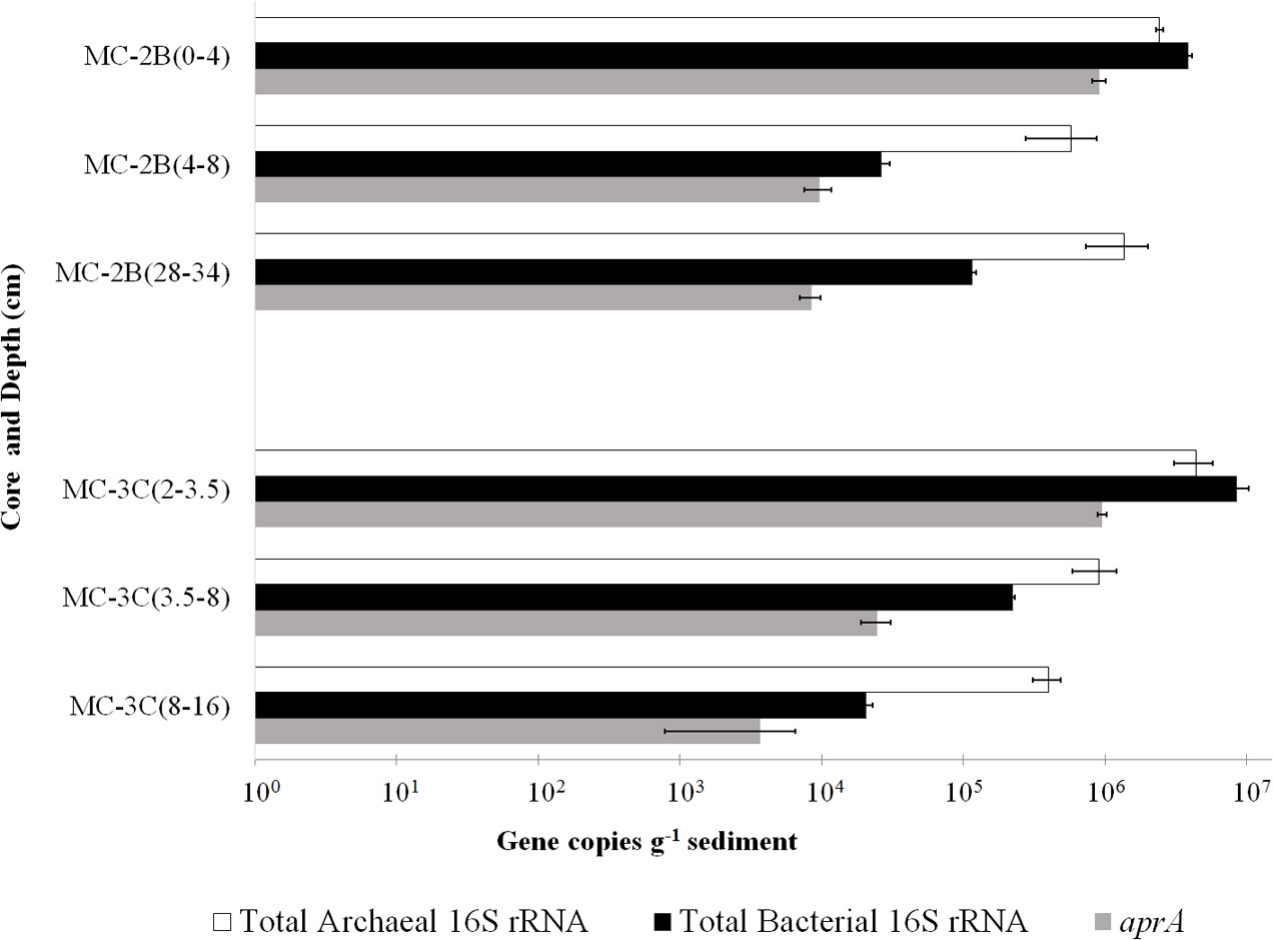
**Q-PCR quantification of bacterial and archaeal 16S rRNA and *aprA* gene copies.** Total bacterial (black bars) and archaeal (white bars) 16S rRNA and *aprA* (gray bars) gene copies from all sediment depths from SLW MC-2B and MC-3C (± SD of technical replicates).

DNA-containing cell abundance in the sediment sample quantified by microscopy was 2.0 × 10^5^ ± 5.1 × 10^4^ cells g^-1^ wet sediment. The microbial cells quantified had morphologies visibly distinct from auto fluorescent mineral grains. Copies of bacterial and archaeal 16S rRNA genes were equally abundant in the surficial sediments, and copy numbers of all three genes decreased with depth (**Table 1**; **Figure 3**). Abundance of total 16S rRNA genes decreased from 6.3 × 10^6^ copies g^-1^ in MC-2B_(0-4__cm)_ and 1.3 × 10^7^ copies g^-1^ in MC-3C_(2-3.5__cm)_ (the two top depths) to 1.5 × 10^6^ copies g^-1^ in MC-2B_(28-34__cm)_ and 4.2 × 10^5^ copies g^-1^ in MC-3C_(8-16__cm)_ (the lower depths; **Table 1**; **Figure 3**). Gene copy numbers of *aprA* decreased from 9.1 × 10^5^ copies g^-1^ in MC-2B_(0-4__cm)_ and 9.6 × 10^5^ copies g^-1^ in MC-3C_(2-3.5__cm)_ to 8.4 × 10^3^ copies g^-1^ in MC-2B_(28-34__cm)_ and 3.7 × 10^3^ copies g^-1^ in MC-3C_(8-16__cm)_ (**Table 1**; **Figure 3**).

### *aprA* GENE

**FIGURE 4:**
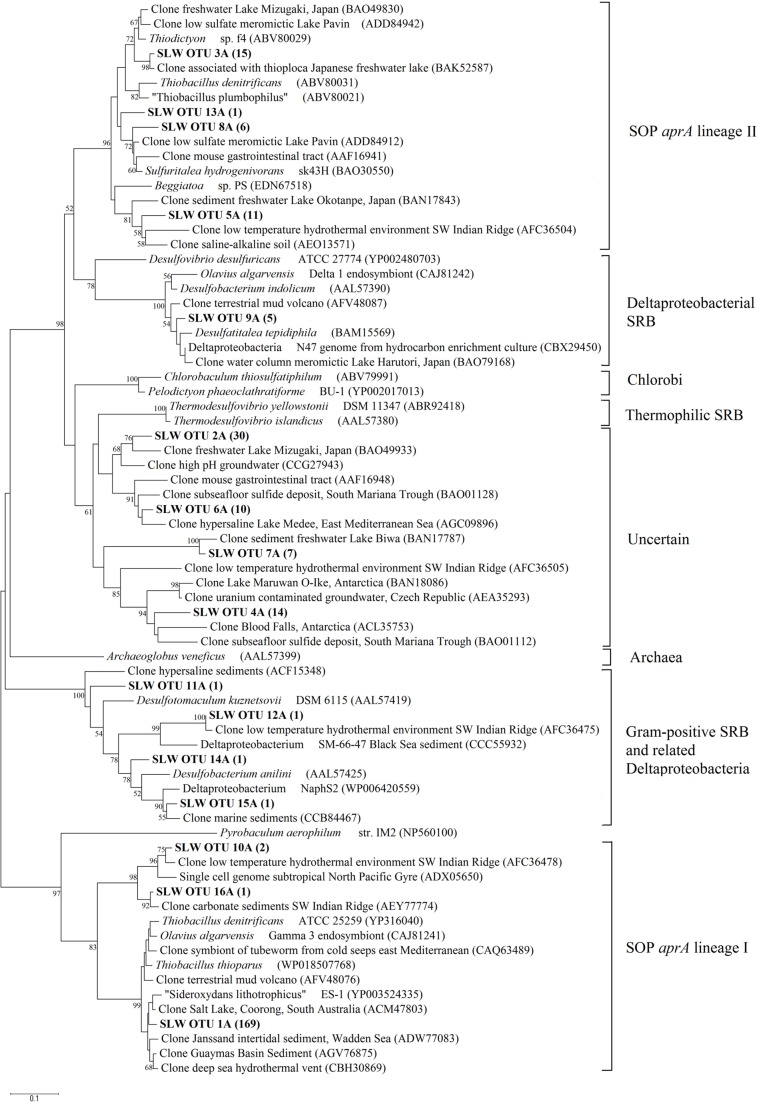
**Phylogenetic tree of SLW sediments *aprA* OTUs.** Neighbor-joining reconstruction of 16 *aprA* sequences from SLW sediments and the most identical *aprA*-containing cultured organisms and environmental sequences. Values at nodes indicate bootstrap support from 1000 replicates. One representative *aprA* sequence from each of the 16 operational taxonomic units (OTUs) was randomly selected and included. SLW *aprA* OTUs are in bold and the total number of sequences obtained within that OTU are in parentheses. Lineage designations on the right are from Meyer and Kuever (2007c). *Pyrobaculum aerophilum* was used as an outgroup reference. Scale bar indicates the branch length corresponding to 0.1 substitutions per amino acid position.

Amplification of *aprA* was detected in all samples (**Table 1**) and a total of 275 *aprA* clones were sequenced from SLW sediments. Both SRP and SOP functional types were present in SLW. The SLW sequences formed 16 distinct OTUs (**Table 4**). *aprA* sequences related to SOP comprised 74% of total *aprA* sequences (**Table 4**). The most abundant *aprA* OTU, 1A, represented 61% of the total sequences and was found in all samples analyzed. OTU1A sequences were affiliated with SOP lineage I, and were 97–94% identical to the *aprA* found in the Betaproteobacterium, “Sideroxydans lithotrophicus” ES-1 (**Table 4**; **Figure 4**). Other SOP-related sequences (OTUs 3A and 13A; combined 6% of total sequences) fell within SOP lineage II and were 95–92% related to *Thiobacillus* spp., including *Thiobacillus denitrificans* and “T. plumbophilus,” and *Thiodictyon* sp. f4 (**Table 4**; **Figure 4**).

**Table 4:** Description of the closest cultured relatives related to SLW *aprA* OTUs and putative sulfur cycle function.

OTUs	% total sequences	Sediment depths observed	Closest cultured representative	% AA identity	Characteristics	Reference
**Sulfur-oxidizing (SOP) – SOP lineage I**						
*1A*	61	All	“Sideroxydans lithotrophicus” ES-1	97–93	Neutrophilic, iron and sulfur oxidizer	Emerson et al. (2013)
*10A, 16A*	1	MC-2B_(0-4, 28-34 cm),_ MC-3C_(3.5-8 cm)_	Single cell genome	93–89	N. Pacific and S. Atlantic Subtropical Gyre at 770 m and 800 m water depth	Swan et al. (2011)
**Sulfur-oxidizing (SOP) – SOP lineage II**						
*3A, 13A*	6	MC-2B_(0-4 cm)_, MC-3C_(2-3.5, 3.5-8 cm)_	“Thiobacillus plumbophilus”	95–92	Mesophilic, aerobic, hydrogen and sulfur oxidizer	Drobner et al. (1992)
			*Thiodictyon* sp.f4	94–93	Photoautotrophic, iron oxidizer	Croal et al. (2004)
*5A,8A*	6	MC-2B_(0-4, 28-34 cm)_, MC-3C_(2-3.5 cm)_	*Sulfuritalea hydrogenivorans*	93–86	Facultative anaerobic autotroph, sulfur oxidizer	Kojima and Fukui (2011)
**Sulfate-reducing (SRP)**						
*9A*	2	MC-3C_(8-16__cm)_	*Desulfobacterium indolicum*	95–94	Anaerobic sulfate reducer from marine sludge	Bak and Widdel (1986)
*11A, 12A*	1	MC-3C_(8-16__cm)_	*Desulfotomaculum kuznetsovii*	88–80	Thermophilic anaerobic heterotrophic sulfate reducer	Visser et al. (2013)
*14A, 15A*	1	MC-2B_(28-34__cm)_, MC-3C_(8-16__cm)_	*Deltaproteobacterium* NaphS2	94–91	Anaerobic sulfate reducer, aromatic compound degradation, from marine sediments	Galushko et al. (1999)
**Uncertain function**						
*2A*	11	MC-2B_ (4-8, 28-34 cm)_, MC-3C_(3.5-8, 8-16 cm)_	*Thermodesulfovibrio yellowstonii*	83–81	Thermophilic heterotrophic, obligate anaerobe, sulfate reducer	Henry et al. (1994)
*7A*	3	MC-2B_(0-4 cm)_, MC-3C_(2-3.5, 3.5-8, 8-16 cm)_	*T. yellowstonii*	71	Thermophilic heterotrophic, obligate anaerobe, sulfate reducer	Henry et al. (1994)
*4A, 6A*	9	MC-2B_(4-8, 28-34 cm)_, MC-3C_(3.5-8, 8-16 cm)_	*T. yellowstonii*	78–69	Thermophilic heterotrophic, obligate anaerobe, sulfate reducer	Henry et al. (1994)
			*Thermodeulfovibrio islandicus*	78–69	Thermophilic sulfate reducer, isolated from hot spring in Iceland	Sonne-Hansen and Ahring (1999)
			*Pelodictyon phaeoclathratiforme*	78–69	Anoxygenic phototrophic sulfur oxidizer, isolated from a meromictic freshwater lake	Overmann and Pfennig (1989)

*SOP lineages and SRP related *aprA* are defined by Meyer and Kuever (2007a).*

Five OTUs (9A, 11A, 12A, 14A, 15A) represented *aprA* sequences (4% of total *aprA* sequences) related to known SRP in samples MC-2B_(28-34__cm)_ and MC-3C_(8-16__cm)_ (**Table 4**). This included *Desulfobacterium anilini* (95–86%), *Desulfatitalea tepidiphila* (95%), and *Desulfotomaculum kuznetsovii* (88–80%; **Table 4**). *aprA* OTUs 9A, 14A, and 15A (collectively comprising 3% of sequences) were most closely related to *Desulfobacterium indolicum* (95–94% identity) and the Deltaproteobacterium strain NaphS2 (94–91% identity). Both of these organisms are known anaerobic sulfate reducers isolated from marine sediments (Bak and Widdel, 1986; Galushko et al., 1999).

Four *aprA* OTUs (2A, 4A, 6A, and 7A) were designated as ‘uncertain’ function and represented 23% of total *aprA* sequences from SLW sediments (**Table 4**). The putative function of these sequences could not be designated with certainty (i.e., involved in either sulfur oxidation or sulfate reduction) because these OTUs align with 83–70% sequence identity to the *aprA* found in *Thermodesulfovibrio* spp. and 79–77% sequence identity to the *aprA* found in the Chlorobi members including *Pelodictyon clathratiforme* (**Table 4**). These OTUs also contained an amino acid insertion (data not shown) at position 311 (numbering after *aprA* in *Allochromatium vinosum*) that is unique to both the *Thermodesulfovibrio* and *Chlorobiaceae aprA* sequences (Meyer and Kuever, 2007b). Detailed molecular analysis has shown that APS genes from *Thermodesulfovibrio* species has been horizontally transferred to these sulfur-oxidizing anoxygenic phototrophic members of *Chlorobiaceae* (Meyer and Kuever, 2007b,c). OTU2A was the second most abundant OTU and represented 11% of total *aprA* sequences in SLW sediments. This OTU had an 83–81% amino acid identity to *Thermodesulfovibrio yellowstonii*, an anaerobic, heterotrophic, sulfate-reducer originally isolated from hydrothermal water (Henry et al., 1994).

### *dsrA* AND *rdsrA* GENES

The primer set targeting a short (221 bp) fragment of *dsrA* amplified in all sediment samples. The longer *dsrAB* (1.9 kb) fragment only amplified in the deeper sediments; MC-2B_(28-34__cm)_ and MC-3C_(3.5-8 and 8-16 cm)_ (**Table 1**) and sequences related to SRP *aprA* were only detected in MC-2B_(28-34__cm)_ and MC-3C_(8-16__cm)_. Fifty-five *dsrA* sequences from samples MC-2B_(0-4 and 4-8 cm)_ and MC-3C_(2-3.5,__3.5-8, and__8-16__cm)_ formed eight distinct OTUs. The majority of these OTUs (80% total sequences) were 78–73% identical to *dsrA* from characterized species in the genera *Desulfotomaculum* and *Carboxydothermus*. The remaining sequences were 88–78% identical to *dsrA* within the Deltaproteobacteria orders *Desulfovibrionales* and *Desulfobacterales* and 99–95% identical to clones from marine sediments (Blazejak and Schippers, 2011).

To increase resolution of SRP phylogenetic diversity, the alpha subunit (255 amino acids) of the *dsrAB* gene from 36 clones collected from MC-2B_(28-34__cm)_ and MC-3C_(3.5-8 and 8-16_ _cm)_ was analyzed. Six distinct OTUs were detected. OTU1D (84% of sequences) represented a deeply branching *dsrA* cluster, with 65–64% sequence identity to *dsrA* from members of the *Desulfotomaculum* genus including *Desulfotomaculum alkaliphilum* and the sulfate-reducing archaeon, *Archaeoglobus veneficus*. OTU1D was 77–75% identical to a *dsrA* environmental clone obtained from various cold marine sediment environments (Harrison et al., 2009; de Rezende et al., 2013). OTU3D (4% of sequences) was most closely related (84% identity) to *dsrA* from the sulfate-reducing Deltaproteobacteria species *Desulfatibacillum alkenivorans*, which is capable of both heterotrophic and chemolithoautotrophic growth (Callaghan et al., 2012) and *Desulfosalsimonas propionica*, a halophilic propionate oxidizer isolated from Great Salt Lake sediments (Kjeldsen et al., 2010). OTUs 2D, 5D, 6D (10% of *dsrA* sequences) were 69–64% identical to Firmicute sequences, including *Desulfurispora thermophila, Desulfotomaculum carboxydivorans,* and *Pelotomaculum propionicum*. *dsrA* OTU4D was 76–72% identical to *Desulfotomaculum carboxydivorans* and *Desulfurispora thermophila*. These organisms are also members of the Firmicutes phyla and are known spore-forming, sulfate-reducers (Parshina et al., 2005; Kaksonen et al., 2007).

Amplification of *rdsrAB* was detected in MC-2B_(0-4__cm)_ and MC-3C_(2-3.5 and 3.5-8 cm)_, but not in MC-2B_(4-8 and 28-34 cm)_, or MC-3C_(8-12__cm)_. Seven unique *rdsrA* OTUs were detected among the 111 *rdsrA* sequences retrieved from SLW sediments. The most abundant OTU, 1R (60% of sequences), was most closely related (83–78% identity) to members of the *Chromatiaceae* family including *Thiorhodococcus drewsii* and *Marichromatium purpuratum*, which are anoxygenic phototrophs capable of oxidizing hydrogen sulfide (Zaar et al., 2003). OTU2R (28% of sequences) was 91–88% identical to *T. denitrificans* and *T. thioparus*. Three OTUs (3R, 4R, and 6R), represented 12% of total *rdsrA* sequences and were 91–80% identical to “Sideroxydans lithotrophicus” ES-1. The remaining OTUs (5R and 7R) represented 2% of total *rdsrA* sequences and were 84–83% identical to *Sulfuritalea hydrogenivorans* sk43H, a facultative anaerobe, mixotroph, and sulfur oxidizer (Kojima and Fukui, 2011).

## DISCUSSION

### ABUNDANCE OF MICROBIAL CELLS, 16S rRNA, AND *aprA* GENE COPIES

Bacterial and archaeal 16S rRNA gene copies varied between cores suggesting SLW sediments were heterogeneous. Our data were consistent with observations from a wide variety of marine sediments (Kallmeyer et al., 2012). Archaeal and bacterial 16S rRNA gene abundances were similar between the two SLW surficial sediment depths studied [MC-2B_(0-4__cm)_ and MC-3C_(2-3.5__cm)_; **Table 1**; **Figure 3**]. However, the number of archaeal 16S rRNA gene copies were higher than bacteria in all other samples; MC-2B_(4-8 and 28-34 cm)_ and MC-3C_(3.5-8 and 8-16 cm)_ (**Table 1**; **Figure 3**). This contrasts with previous results from SLW surficial sediments. Christner et al. (2014) detected low relative abundance of archaeal phylotypes in the SLW water column and surficial (0–2 cm) sediments (3.6 and 0.3%, respectively). This discrepancy could be due to primer bias; the primers used in our Q-PCR analysis detect a wider range of archaea [according to *in silico* analysis using TestPrime 1.0 application (Klindworth et al., 2012) and the SILVA SSU r119 RefNR database (Quast et al., 2013^7^)]. Alternatively it could be due to heterogeneity. Compiled cell density data (based on both Q-PCR and fluorescence *in situ* hybridization) from 65 studies of marine sediments showed that abundance of archaea and bacteria vary and dominate at different sites throughout the global ocean (Lloyd et al., 2013).

16S rRNA gene copy number cannot be directly converted into biomass, however, by accounting for average 16S rRNA copy number within sequenced genomes, gene copy number can be used as a proxy for total cells. A survey of the currently finished microbial genomes in JGI IMG (July 2014) indicated that on average there are 4.04 rRNA gene copies per bacteria cell and 1.64 copies per archaeal cells (Markowitz et al., 2014). Based on these values and averaging the Q-PCR results for all depths, we estimated a microbial abundance of 1.6 × 10^6^ cells g^-1^ wet sediment, which is an order of magnitude higher than our microscopy counts. Numerous protocols have been developed for quantification of microbial cells in sediments using fluorescent nucleic acid stains (Klauth et al., 2004; Kallmeyer et al., 2008; Morono et al., 2013). However, quantification remains challenging due to auto-fluorescent properties of sediment particles, non-specific binding of nucleic acid stains, or particle blocked microbial cells (Kepner and Pratt, 1994). Lloyd et al. (2013) determined that 16S rRNA gene quantification and microscopy cell counts vary and cannot be consistently correlated.

Functional gene abundances relative to total 16S rRNA gene copies have been used to estimate population densities (Henry et al., 2006). Copies of *aprA* in SLW sediments represented 7.3% [MC-2B_(0-4__cm)_] and 14.5% [MC-3C_(2-3.5__cm)_] of total 16S rRNA gene copies in the top depths. These percentages decreased to 1.6% [MC-2B_(4-8)_] and 2.2% [MC-3C_(2-3.5)_] in the middle depths, and 0.6% [MC-2B_(28-34__cm)_] and 0.9% [MC-3C_(8-16__cm)_] in the deeper depths (**Table 1**). The *aprA* primers used for this study are universal for bacteria and archaea, however no archaeal *aprA* were detected. If we compare *aprA* abundance relative to total bacterial 16S rRNA, 18% ± 9.9 of the total bacterial population is represented (**Table 1**). This proportion is higher than reports from 40 m below the Peru Margin seafloor, where *aprA* represented 0.5–1% of bacterial 16S rRNA gene copies (Blazejak and Schippers, 2011). While the abundance of *aprA* containing cells varied in our samples, they appear to comprise a large portion of the community.

### COMMUNITY STRUCTURE, FUNCTION, AND DIVERSITY IN SLW SEDIMENTS

**FIGURE 5:**
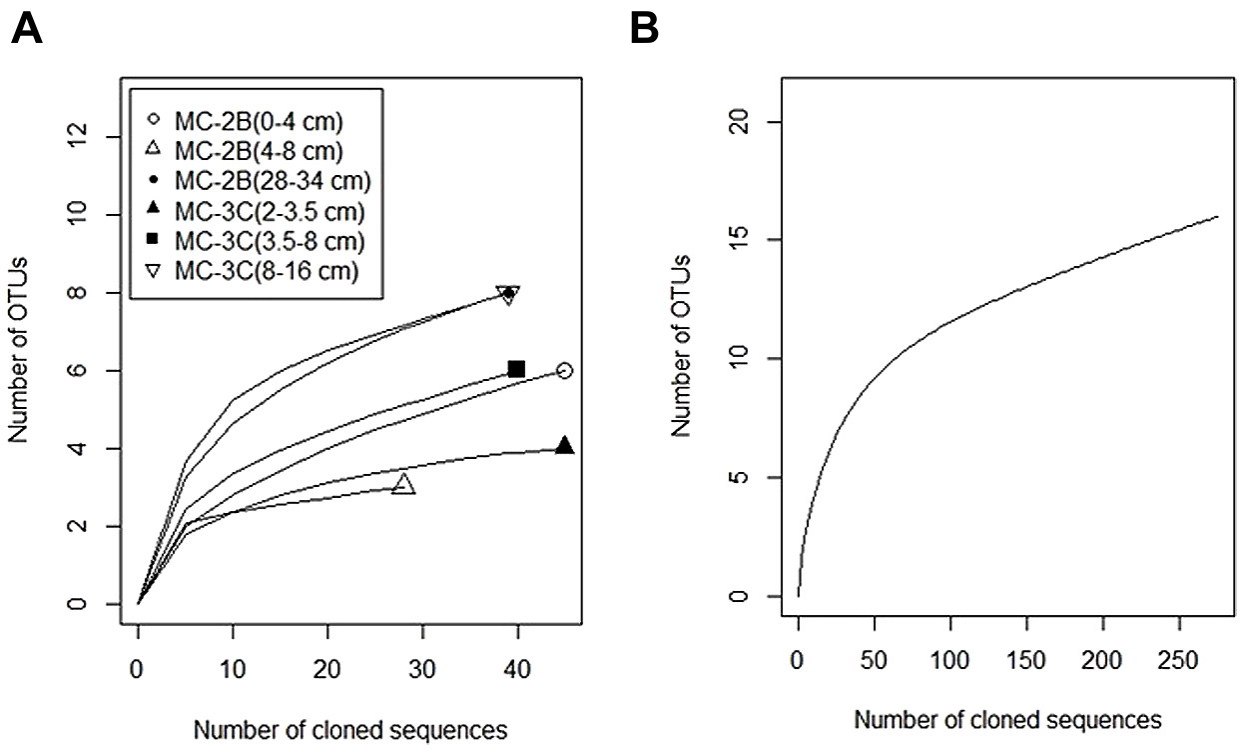
**Distribution of *aprA* sequences from SLW sediment cores MC-2B and MC-3C among putative sulfur-cycling lineages.** Sulfur cycling lineages as functional categories defined by Meyer and Kuever (2007a). Blue represents sulfur-oxidizing prokaryote lineages I and II, black represents sulfate-reducing lineages, and gray represents sequences of uncertain function. Percentages represent the number of sequences within the designated lineage out of the total *aprA* sequences obtained from each depth. The total number of sequences obtained for each depth are listed in **Table 3**.

The presence of *aprA*, *dsrAB*, and *rdsrAB* in sediments from SLW indicated the microbial community had the genetic potential to mediate a variety of sulfur transformations (**Table 1**). Functional lineages of *aprA* (e.g., oxidation, reduction, or uncertain function) varied with depth in both sediment cores (**Figure 5**) although sulfur-oxidizing *aprA* was present in all depths analyzed. SOP-like sequences were all related to autotrophs or facultative autotrophs, supporting the potential for sulfur driven chemosynthesis in the top 34 cm of SLW sediments (**Table 4**; **Figure 5**).

**FIGURE 6:**
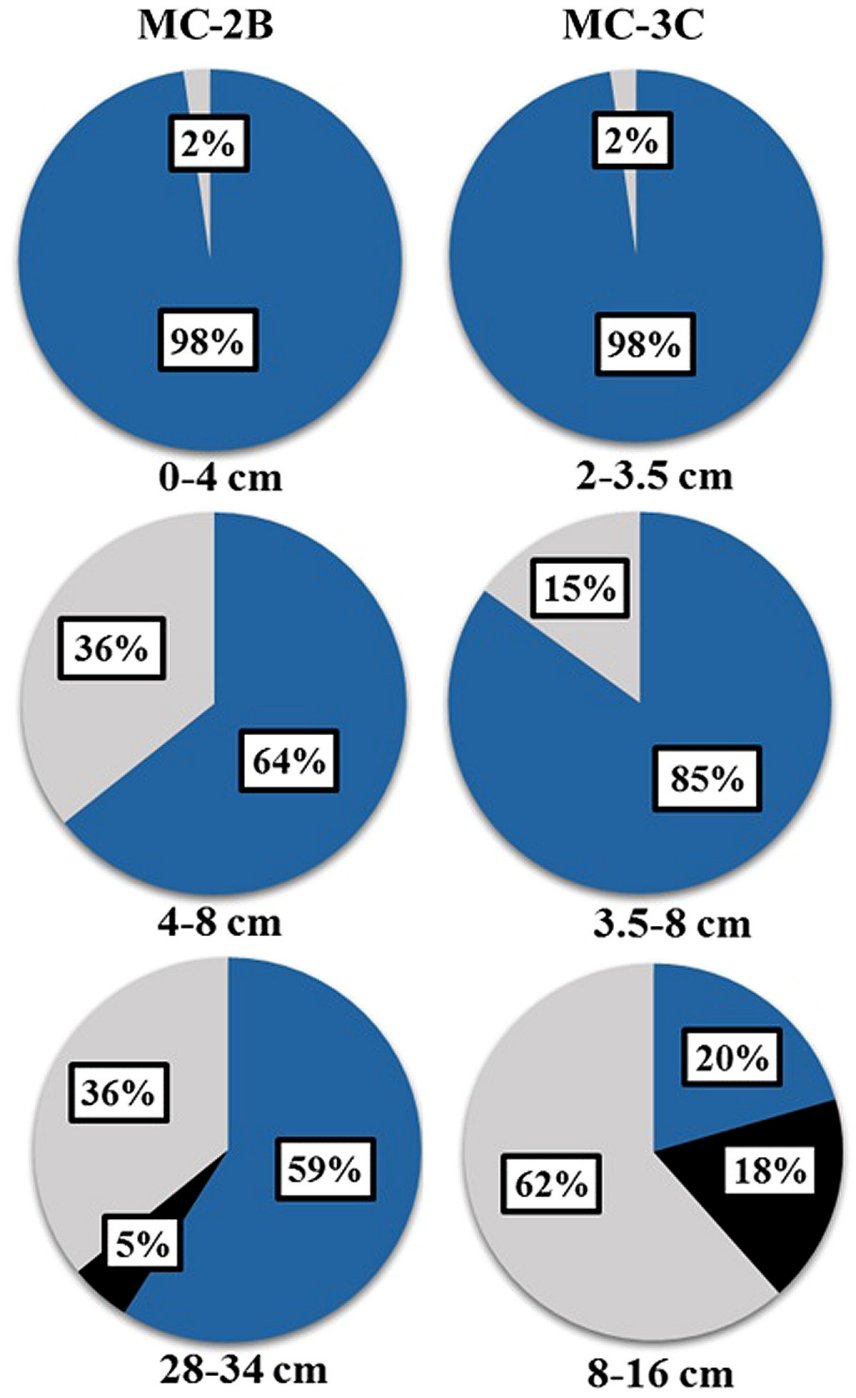
**Rarefaction curves of *aprA* in SLW sediments. (A)** Individual depths from sediment cores MC-2B and MC-3C. **(B)** Total *aprA* sequences from all depths and cores.

Diversity measurements can be used to compare depth profiles or different environments. The Shannon-Weaver diversity index (*H′*) varied from 0.63 to 1.82 suggesting heterogeneity in sediment samples (**Table 3**). Sulfur-oxidizing *aprA* sequences were dominant in the top sediment depths (98% of total *aprA* sequences; **Figure 6**), and had low diversity (**Table 3**). The abundance of *aprA* sequences related to sulfur oxidizers decreased with depth (**Figure 5**), but overall *aprA* sequence diversity was higher at deeper depths; MC-2B_(28-34__cm)_ and MC-3C_(8-16__cm)_, *H′* values, 1.62 and 1.82, respectively. The diversity in SLW is lower than other, mineral rich environments such as a low temperature hydrothermal field (*H′* = 2.3–3.1) with high concentrations of Fe, Si, and Mn (Li et al., 2013) or organic-rich environments such as Brazilian mangrove sediments (H′ = 1.9–2.6; Varon-Lopez et al., 2014). SRP-related *aprA* sequences were only detected in MC-2B_(28-34__cm)_ and MC-3C_(8-16__cm)_ (**Table 4**; **Figure 5**), suggesting a shift in the sulfur cycling community structure with depth. The total SOP community in SLW was likely undersampled (**Table 3**; **Figure 6**) since the alternative oxidation pathways (e.g., the SOX enzyme system), present in many SOP (Meyer et al., 2007), were not addressed in this study.

### SULFUR-OXIDIZING PROKARYOTES IN SLW SEDIMENTS

Combined *aprA* and *rdsrA* analyses indicated that the dominant sulfur oxidizer in SLW was related to “Sideroxydans lithotrophicus” ES-1. Strain ES-1 was originally isolated from groundwater in Michigan and has been characterized as a neutrophilic, microaerophilic, iron oxidizer also capable of oxidizing reduced sulfur compounds such as thiosulfate and iron sulfide (Emerson and Moyer, 1997; Emerson et al., 2013). *Thiobacillus* spp. and *Sulfuritalea hydrogenivorans* sk43H, known chemoautotrophs (Kelly, 1982; Drobner et al., 1992; Kojima and Fukui, 2011), were also detected in both *aprA* and *rdsrA* clone libraries. The 16S rRNA gene survey on MC-2B_(0-2__cm)_ sediments found that a “Sideroxydans”-like phylotype was most abundant (12.7% total sequences) and a *Thiobacillus*-like phylotype (6.1% total sequences) was also present (Christner et al., 2014). Our results are also consistent with findings from other subglacial systems. For example, the 16S rRNA gene sequence identified as “Sideroxydans lithotrophicus” from SLW (Christner et al., 2014) was 99% identical to a clone from sediments beneath the Kamb Ice Stream (Lanoil et al., 2009). Analysis of samples from Robertson Glacier in Canada revealed that a “Sideroxydans” sp. comprised 12% of 16S rRNA transcripts (Hamilton et al., 2013) and is likely the dominant mediator of chemosynthesis via pyrite oxidation in this environment (Boyd et al., 2014). Our *aprA* sequence data, in combination with previously published 16S rRNA gene data, strongly support the notion that sulfur oxidation is a dominant metabolic process in SLW sediments, largely facilitated by a “Sideroxydans*”*-like organism. These organisms may play a key role in subglacial microbial ecosystems, perhaps as primary producers.

The rDSR pathway may facilitate a chemosynthetic lifestyle for some SOP in SLW. The most abundant *rdsrA* OTU (1R) from SLW sediments was most closely related to cultured phototrophs. However, few surveys of rDSR diversity in environmental samples have been published, which is in contrast to APS and DSR (Loy et al., 2009). Experiments on isolates show rDSR is necessary for the oxidation of intracellular sulfur globules, temporary storage reservoirs that are formed during the oxidation of sulfides in many SOP (Dahl et al., 2005; Holkenbrink et al., 2011). Physiological studies on the function of rDSR in chemoautotrophs, although limited (Loy et al., 2008), suggests rDSR may be important in energy gain from elemental sulfur in the dark ocean (Anantharaman et al., 2014). It has been suggested that the rDSR/reverse APS reductase pathway for sulfur oxidation is more efficient in environments with low sulfide concentrations (Frigaard and Bryant, 2008; Gregersen et al., 2011; Holkenbrink et al., 2011). Energetic efficiency might convey a competitive advantage to microorganisms in SLW sediments making the rDSR pathway preferred for sulfur oxidation.

### SULFATE REDUCTION IN SLW SEDIMENTS

Functional gene data and activity assays both indicated that SRP are present in SLW sediments, although SRR were extremely low (**Table 1**; **Figure 2**). Analysis of 16S rRNA gene libraries also indicated that known sulfate-reducing taxa were not abundant members of the water column (0.1% OTUs) or the MC-2B_(0-2__cm)_ (0.02%) sediment community (Christner et al., 2014). Sulfate reduction was not detected in the SLW water column, likely due to the presence of oxygen. The low measured rates in the sediments could also be due to rapid reoxidation of reduced sulfur. If reduced sulfur generated by SRP activity was reoxidized before it could be scavenged by the zinc trap in our experimental design, our rates would be an underestimation; FeS, FeS_2_ and S^2-^ are not released from solution until the experiment is terminated by passive extraction. A similar process, where reduced sulfur was quantitatively reoxidized to sulfate, described as a catalytic sulfur cycle, was observed in the Blood Falls subglacial brine based on the isotopic composition of sulfate δ^34^S and δ^18^O (Mikucki et al., 2009). This type of sulfur cycle has also been detected in marine oxygen-minimum zones and is referred to as a cryptic sulfur cycle (Canfield et al., 2010). Low rates (0.2–1.0 pmol cm^-3^ d^-1^), of the same order of magnitude as SLW, were measured in marine sediments at 3–5 m depth, which were 4–5 orders of magnitude less than those measured at the surface (Holmkvist et al., 2011). Rapid turnover of reduced sulfur may be an economical strategy for energy gain in deep subsurface environments.

*dsrA* was detected in all samples with measureable sulfate reduction; although the distribution of *aprA* and *dsrAB* were less consistent (**Table 1**; **Figure 2**). *dsrAB* abundance may have been below the detection limit, or not detectable with the primer set used. While no *aprA* sequences related to known SRP were detected in MC-2B_(0-4 and 4-8 cm)_ and MC-3C_(2-3.5 and 3.5-8 cm)_, the small amounts of reduced sulfur detected in our SRR experiments from these depths could have been generated by organisms carrying the *aprA* sequences of uncertain function, (i.e., those related to both *Thermodesulfovibrio* and members of *Chlorobiaceae).* For example, *aprA* OTU 4A, amplified from MC-2B_(4-8__cm)_ and MC-3C_(3.5-8__cm)_ (**Table 4**), was 83% identical to a clone from Blood Falls, where sulfate reduction occurs (Mikucki et al., 2009), and both were 74–72% identical to *Thermodesulfovibrio* spp, which are known SRP.

MC-2B_(28-34__cm)_ and MC-3C_(8-16__cm)_ contained both *aprA* and *dsrA* sequences related to *Desulfotomaculum* spp. and the Deltaproteobacteria family *Desulfobacteraceae*. It has been argued that *Desulfotomaculum* spp. play an important ecological role in subsurface environments because they are metabolically plastic. They can grow under a range of sulfate concentrations, use diverse organic substrates, are capable of autotrophy, participate in syntrophic relationships with methanogens, and can form endospores (Imachi et al., 2006; Aüllo et al., 2013). The *dsrA* sequences from SLW sediments could represent active microorganisms adapted to freshwater, as related sequences have been found in other low sulfate environments (Kondo et al., 2007). Alternatively, given the low sequence similarity to characterized SRP, *dsrA* detected in this study could represent novel SRP lineages. Most *Desulfobacteraceae* isolates have been found in marine and hypersaline sediments (Foti et al., 2007; Kjeldsen et al., 2007), however *aprA* sequences related to this group have also been detected in freshwater lakes (Biderre-Petit et al., 2011), including ice-covered lakes Oyako-Ike and Skalle O-Ike in Antarctica (Watanabe et al., 2013). These OTUs could represent organisms that provide reduced sulfur compounds to SOP at deeper sediment depths and use alternative electron acceptors such as nitrate or ferric iron in the absence of oxygen. The concentration of nitrate was higher in the upper 2 cm of SLW sediments (9.1 μM) compared to the water column (0.8 μM; Christner et al., 2014), suggesting nitrate is available for microbial reduction. Sulfide oxidation can also be coupled to ferric iron reduction (Schink, 2006), although ferric iron concentrations for SLW have not been processed.

## CONCLUSION

Combined analyses of the functional genes *aprA*, *dsrA*, and *rdsrA*, in concert with measureable rates of sulfate reduction under anaerobic conditions revealed a diverse community capable of sulfate reduction and sulfur oxidation in SLW sediments. Functional gene OTUs in this study represented groups that encompass a broad range of physiological traits. While some OTUs were related to previously documented species from environments including marine sediments, groundwater, and freshwater lakes, many of the OTUs represent novel lineages whose function is not yet known. Our results further support the fact that Antarctic subglacial aquatic environments host a diverse microbial ecosystem that remains inadequately studied. These data provide new insight into the structure of microbial communities in subglacial environments.

The presence of chemosynthetic sulfur oxidizers in SLW surface sediments reinforces previous reports of sulfur oxidation at subglacial sediment-water interfaces (e.g., Tranter et al., 2002; Skidmore et al., 2005; Lanoil et al., 2009; Hamilton et al., 2013) and supports the importance of dark CO_2_ fixation in subglacial environments (Boyd et al., 2014). We also provide estimates of sulfate reduction rates from samples below the West Antarctic Ice Sheet. Rapid retreat of the grounding line and eventual collapse of the Ross Ice Shelf and West Antarctic Ice Sheet will expose the subglacial ecosystem to marine conditions, as has happened in the past (Mercer, 1978; Scherer et al., 1998). Understanding the structure and function of subglacial microbial communities can help predict the ecological impact of ice sheet thinning or retreat to the proglacial ecosystem.

## AUTHOR CONTRIBUTIONS

Alicia M. Purcell and Jill A. Mikucki designed and conducted the experiments, analyzed the data and wrote the manuscript. Dhritiman Ghosh, Andrew C. Mitchell, John C. Priscu, Mark L. Skidmore, and Alexander B. Michaud assisted with data analyses. Amanda M. Achberger and Brent C. Christner provided input on molecular analyses. Reed Scherer provided sediment descriptions. All authors contributed to sample acquisition, manuscript revisions, and approved the final submitted version.

## Conflict of Interest Statement

The authors declare that the research was conducted in the absence of any commercial or financial relationships that could be construed as a potential conflict of interest.

## Footnotes

1 http://www.wissard.org

2 http://www.arb-silva.de

3 http://www.mbio.ncsu.edu/bioedit/bioedit.html

4 http://toolkit.tuebingen.mpg.de/blastclust

5 http://blast.be-md.ncbi.nlm.nih.gov/Blast.cgi

6 http://www.jennajacobs.org/R/rarefaction.html

7 http://www.arb-silva.de

## Abbreviations

SLW: Subglacial Lake Whillans
WIS: Whillans Ice Stream
SOP: sulfur-oxidizing prokaryotes
SRP: sulfate-reducing prokaryotes
APS: adenosine-5′-phosphosulfate reductase
DSR: dissimilatory sulfite reductase
rDSR: reverse dissimilatory sulfite reductase
